# Can Green Economy and Ecological Welfare Achieve Synergistic Development? The Perspective of the “Two Mountains” Theory

**DOI:** 10.3390/ijerph19116460

**Published:** 2022-05-26

**Authors:** Lindong Ma, Yuanxiao Hong, Xihui Chen

**Affiliations:** 1 School of Management, Zhejiang University of Technology, Hangzhou 310023, China; malindong@zjnu.edu.cn; 2 Xingzhi College, Zhejiang Normal University, Jinhua 321004, China; hyx021@zjnu.edu.cn; 3 Business & Tourism Institute, Hangzhou Vocational & Technical College, Hangzhou 310000, China

**Keywords:** green economic efficiency, ecological welfare performance, coupling coordination degree, Tobit model, “Two Mountains” theory, Zhejiang Province, China

## Abstract

China’s high-speed economic growth and severe environmental problems have resulted in a poor Environmental Performance Index and have affected China’s sustainable development and ecological welfare improvement. Therefore, exploring whether there is a certain relationship between the two and their influencing factors is an important way and a breakthrough to solve the problems regarding green economic progress and ecological welfare enhancement. To this end, by using the undesirable slack-based measure (SBM) model, this paper measures the ecological welfare performance and the green economic efficiency of 11 cities in Zhejiang Province, China, from 2000 to 2019. Through the methods of spatiotemporal evolution, coefficient of variation, coupling coordination degree, and the Tobit model, we found that: (1) The development trend of urban green economic efficiency and ecological welfare performance were both in a “U” shape that first fell and then rose; (2) The coupling coordination degree between green economic efficiency and ecological welfare performance showed a wave-like upward trend as a whole and most cities have entered a more advanced coupling coordination stage during the study period. The coefficient of variation revealed a downward trend; (3) The urbanization level, industrial structure, and government investment can promote the regional coordinated development, while the industrialization degree and the opening level had a negative impact on it; (4) The “Two Mountains” theory was beneficial to the improvement of regional urban green economic efficiency and ecological welfare performance and their coordinated development both in theory and practice. Finally, according to the findings, we offer relevant suggestions on making good use of the country’s preferential policies and informatization means from the perspective of the regional coordinated development.

## 1. Introduction

In the past few decades, China’s economy has grown rapidly, and its GDP has ranked second in the world for many consecutive years [1]. The sustained, stable, and high-speed economic growth has greatly improved the country’s economic level and economic strength, but it has also paid a heavy ecological price. Issues such as energy shortage and environmental pollution have increasingly become the main bottlenecks restricting economic and social development. Yale University assessed the Environmental Performance Index (EPI) in 180 countries in 2020, and China’s 120th place with a score of 37.3 is a good illustration of this. How to properly handle economic growth, ecological protection, and the improvement of people’s livelihood and well-being is a common challenge faced by human society [2]. For this purpose, as early as 2005, Xi Jinping, who was then secretary of the Zhejiang Provincial Party Committee, creatively proposed the “Two Mountains” theory based on the concept of strengthening ecological civilization and green development, that is, “Lucid waters and lush mountains are invaluable assets”. It has played an important role in the naturalization of various habitats [3]. Especially since the 18th National Congress of the Communist Party of China, the Central Committee of the Communist Party of China has paid more attention to the construction of an ecological civilization and green development. China’s green development is ushering in good policy opportunities. At the same time, the green economy development has been highly valued by countries and regions worldwide [3], will become the main way of economic and social development in the future, and is an important way to achieve sustainable development [4] and a “booster” to achieve high-quality development [5]. A good ecological environment and welfare supply are effective solutions to people’s current demand for a better life to some extent. On the one hand, green economic efficiency is a comprehensive reflection of the green economy and the “key pulse” of green economic development [5]. On the other hand, ecological welfare performance can truthfully indicate the local ecological governance level and people’s happiness [6], which is an important reflection of the achievement of people’s goal of sustainable development [1]. Another very important aspect is that the Zhejiang Province is the birthplace of the “Two Mountains” theory as well as the vanguard of the country and the frontier of common prosperity. In addition, due to Zhejiang’s social conditions, such as the urban-rural gap, regional development, and affluence, as well as its geographical conditions of “seven mountains, one water, and two fields”, it is quite similar to a scaled-down version of China so that it is suitable to form replicable and generalizable experiences. Therefore, revealing the coupling mechanism, relationship and influencing factors of the two through the typical case of the Zhejiang Province is not only a quantitative verification of the “Two Mountains” theory, but also an inherent requirement for implementing high-quality development. It is also an effective solution to the basic contradictions in social and economic development.

Green economic efficiency is an important indicator for evaluating the production efficiency of a country or region by considering resource inputs and environmental costs. It reflects the efficiency of utilizing natural resources and reducing environmental pressure in the process of pursuing economic benefits [7]. It can be said that green economic efficiency is the essence of green economic development [8]. The current research on green economic efficiency mainly focused on the following main aspects: Firstly, the index construction and measurement of green economic efficiency were carried out, and the spatial and temporal characteristics of the study area were analyzed [8,9,10,11]. Due to the lack of a unified measurement index system and calculation method and the differences in economic development and ecological environment in various regions, the results were relative and inconsistent. Secondly, the relationship between green economic efficiency and industries was discussed, concerning different industries [10,12], industrial structure and transfer [13,14], and industrial agglomeration [10,15]. Thirdly, the mutual choice and integration between the regional government and green economic efficiency were deeply discussed and studied, including the hot issue of environmental regulation [16,17,18,19], fiscal decentralization [20], local government competition [21], and policy uncertainties and market integration [22,23]. Other influencing factors of green economic efficiency studied were as follows: technology imports and innovation [24,25,26], human capital [27], urbanization [28], resource structure and consumption [29,30,31], foreign direct investment [32], digital economy [33], etc. Many useful conclusions have been drawn. The starting point of studying green economic efficiency is for the sustainable, sound, and healthy development of the economy. However, whether sustainable and healthy development of the economy can effectively promote ecology and welfare is less involved at present. Therefore, its relationship with ecological welfare performance needs to be further explored and enriched.

Humans currently face a problematic ecological dilemma regarding economic growth. It is difficult to meet human needs by only studying economic growth created by artificial costs, and all countries need to pay attention to the task of improving the human welfare level under the constraints of the ecological environment from the perspective of sustainable development [34]. Ecological welfare performance is the ratio of social welfare value to the physical quantity of ecological consumption, which reflects the welfare output per unit of ecological consumption, and can truthfully reflect the local ecological governance level and people’s happiness [6]. From the existing literature, the content mainly focused on, first, the measurement and evaluation of ecological welfare performance [35]. For example, Rong Wang and Yue Feng [36] evaluated China’s ecological welfare performance (2006–2018) from a static and dynamic perspective. Xiao Liming et al. [37] analyzed the urban ecological welfare performance pattern and spatial convergence in the Yellow River Basin. A similar analysis was also conducted at the provincial level [38], in the Yangtze River Delta [39] and other major cities in the country [40]. Although the focuses were slightly different, their contents were mainly about the evaluation and spatial distribution of ecological welfare performance. Second, is the research on the influencing factors of ecological welfare performance and their relationship. It mainly involves the relationship between ecological welfare performance and economic growth [41,42], urbanization [43], environmental regulation, industrial structure [44], technological innovation, foreign investment, etc. (Behjat [42] revealed that the relationship between economic growth and ecological welfare performance was positive and statistically significant). The above studies have greatly enriched the breadth and depth of ecological welfare performance and have drawn many beneficial conclusions, which have laid a foundation for further research. However, there is no discussion on its relationship with the green economic efficiency system and the influencing factors, which represent the future development trend.

In terms of research methods, there are two main categories, stochastic frontier analysis (SFA) and data envelopment analysis (DEA), measuring green economic efficiency and ecological welfare performance at their respective levels. The SFA method needs to set parameters in advance, while the DEA method has the advantage that there is no need to set a specific production function, the indicators being not affected by dimensions, and being capable of dealing with multiple inputs and multiple outputs. Therefore, it was widely favored in measuring the ecological welfare performance [37], for example, Li Chengyu et al. measured China’s interprovincial ecological welfare performance based on the unexpected output SBM. Regarding the measurement of green economic efficiency, scholars from other countries mostly used the DEA model [45], which was also widely used in China [46,47,48]. Accordingly, this paper also adopts the undesired output SBM model that was based on the DEA method. In terms of research scope selection, they were mainly based on the national (provincial level) level and regional level (prefecture-level city level). Therefore, referring to previous studies, this paper also uses DEA undesirable output SBM method to evaluate the respective value of each system and learn their development trend from it.

The above research provides an important basis for this study, and the following aspects are further enriched and improved in this paper. First, green innovation efficiency focuses on the future sustainable development of the economy and society, while ecological welfare performance is the perceived “benefits” brought to the people. The study of their temporal and spatial development process provides a reference for the implementation of Zhejiang’s high-quality development for achieving a Common Prosperity Demonstration Zone. It also verifies the effect of the “Two Mountains” theory at the regional level in time and space dimensions. Secondly, it deeply analyzes and excavates the dynamic evolution law of the coupling coordination degree between ecological welfare performance and green economic efficiency and its influencing factors, enriching relevant theories and providing a reference for practice. Finally, it provides a reference for the better implementation of Zhejiang’s high-quality development and the realization of the Common Prosperity Demonstration Zone and provides suggestions for the development of similar regions across the country and the world.

This paper has the following structure: First, an analysis of the coupling mechanism of green economic efficiency and ecological welfare performance. Secondly, the introduction of data and methods. Thirdly, the analysis of results and the explanation of influencing factors. Finally, it comes to the discussion, conclusion, and policy recommendations.

## 2. Theoretical Analysis and Research Hypothesis

**Figure 1:**
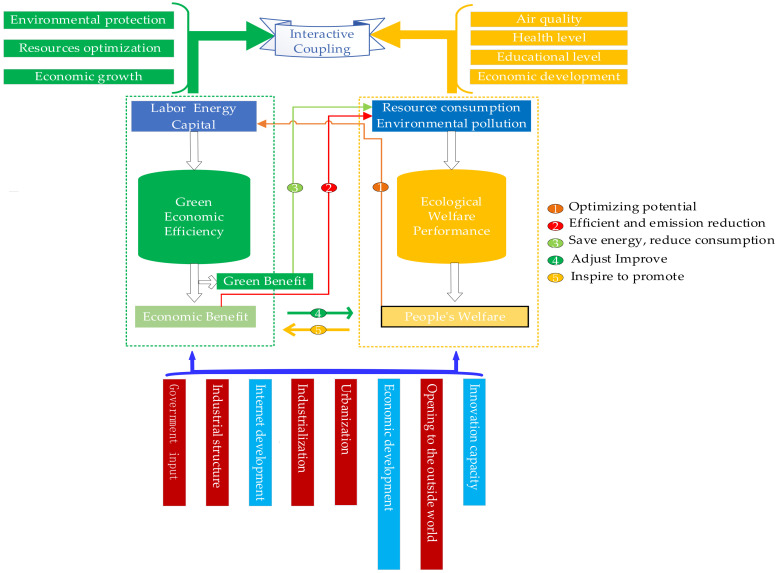
The coupling and driving mechanism of green economic efficiency and ecological welfare performance.

Green economy is a new economic development model that realizes the balance between economic growth and resource environment optimization [49]. Green economy efficiency is an efficiency-oriented green economy theory for improving the efficiency of the economic system. Green economy efficiency is also the internal driving force for the improvement of ecological welfare performance. The impact of green economic efficiency on ecological welfare performance is mainly reflected in two aspects: economic development and green development (see Figure 1). Economic development is the support of social progress and ecological civilization construction [28], and green development is the core of constructing the theoretical framework of ecological welfare performance [37]. With the emphasis on green development and the economy entering a new normal in recent years, the pollutant emissions per unit of GDP declined and green components increased. It will inevitably be accompanied by more stringent requirements on corporate and social pollutants emissions. To this end, enterprises must transform and upgrade to construct a “two-type” society, which helps to optimize the input indicators of the ecological welfare performance [50]. At the same time, due to the reduction in pollutant emissions per space unit, the pressure on the regional ecology has been alleviated, and the source of PM_2.5_ causes has been reduced [51], thereby the air quality has been further improved. In addition, the enhancement of economic strength driven by economic development can effectively solve the problems of education, medical care, and basic livelihood projects that people care about. This will greatly enhance the happiness and well-being of residents. Therefore, the green economy development efficiency has ecological welfare effects. However, the rapid economic development has also brought environmental problems. High investment, high consumption, and high pollution will inevitably have a significant negative impact on the regional environment. The dual role of economic development cannot be viewed one-sidedly. Thus, the level of industrialization development, regional industrial structure, and urbanization will affect the coordinated development of the two systems.

The improvement of ecological welfare performance is the core of solving the “dilemma” regarding economic growth, ecological protection, and the improvement of people’s livelihood and well-being. We should face up to the evolution trend of ecological welfare performance and realize the development mode transformation from “incremental” to “quality improvement” [47]. The ecological welfare performance improvement can effectively help to improve green development and economy, thus further improving the green economy efficiency. At the same time, due to the improvement of ecological welfare performance, people’s education level and medical security were further improved accordingly, which not only roared the people’s consumption potential unleashed without worries, but also showed the people’s objective needs in pursuit of better well-being. In turn, it will further promote the steady progress of the economy and society to achieve high-quality development.

From a micro perspective, the closed enterprises will be further transformed and upgraded, and the environmental quality and economic development level will be steadily improved and go hand in hand accordingly. In this interactive promotion process, residents will have a better education level due to the improvement of education quality under the Human Development Index (HDI) welfare framework. The joining of high-quality talents has improved the quantity and quality of employees [50], which is conducive to optimizing the regional human resources structure, thereby injecting unlimited human resource potential into developing the regional green economy. No matter whether the improvement of public welfare is in education or medical care, it requires the support of the government and the society to carry out the second or even third wealth distribution and transfer. So, the role of government transfer payments cannot be ignored. Therefore, the two systems of green economic efficiency and ecological welfare performance have formed a benign and mutually reinforcing causal cycle under the joint action of nature, economy, and society. The interaction, complementarity, and close linkage of the two systems can achieve a benign state of global resource optimization and coordinated progress, providing an internal driving force for the high-quality development of the region.

## 3. Methodology and Data

### 3.1. Study Area

**Figure 2:**
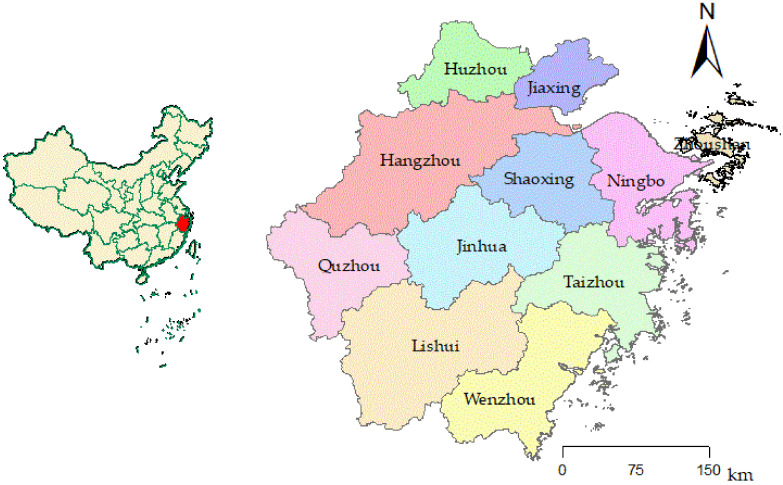
City map of the Zhejiang Province and its location in China.

The Zhejiang Province (118°01′~123°10′ E, 27°02′~31°11′ N) is located on the southeast coast at the southern wing of the Yangtze River Delta and borders Shanghai and Jiangsu in the north. The terrain is dominated by mountains and hills, known as “seven mountains, one water, two fields”. With a land area of 105,500 km^2^ and a population of 64,567,500 (data from the 7th national census), it is one of the small provinces in China. In 2021, the gross regional product (GDP) of the Zhejiang Province was CNY 73,516 billion, an increase of 8.5% over the previous year, with the ratio of the three industries being 3.4:43.6:53.0. The per capita GDP was 113,900 yuan (USD 17,666 at the annual average exchange rate). Zhejiang is one of the provinces that has the smallest gap in regional economic development. It is the birthplace of the “Two Mountains” theory and the exploration site of high-quality development for constructing a “Common Prosperity Demonstration Zone”. The research into it has good representativeness and is of special significance. It is regarded as a weathervane and a foreword position for grasping the future development direction of China. Therefore, this paper makes an in-depth analysis and exploration of it. By the end of 2021, there were 11 prefecture-level cities under the jurisdiction of the Zhejiang Province. ArcGIS 10.6 was used for spatial analysis, as shown in Figure 2.

### 3.2. Indicator System and Data Description

#### 3.2.1. Green Economic Efficiency

According to the existing research on green economic efficiency and the fact that China has entered a new normal, the environmental carrying capacity has reached or is close to the upper limit [52]. Considering the existing research results [11,14,19,22,32,33,53] and the availability and accuracy of data, energy, labor, and capital were selected as input elements. Meanwhile, regional GDP, industrial wastewater, industrial sulfur dioxide, and industrial smoke and dust were chosen as outputs. The specific index variable definitions are shown in Table 1.

#### 3.2.2. Ecological Welfare Performance

The selection of indicator variables for ecological welfare performance followed the principles of scientificity, systematization, and operability. Based on a systematic review and reference to existing research results [1,36,42,43,54], resource consumption and environmental pollution were selected as input indicators (see Table 2). Under the HDI framework, air quality was added as an output variable for ecological welfare, in addition to the economy, education, and health care. As the most populous developing country, the accelerated trend of deterioration in the atmospheric environment in China has been obvious in recent years [55]. Due to the serious air pollution, the worsening atmospheric environmental quality has become increasingly prominent and has caused great harm to people’s production, life, and health [56,57]. In comparison, the actual solid waste treatment and recycling rate of the Zhejiang Province were both above 92% (the data are from the Statistical Yearbook of Zhejiang Province, similar as follows), and the compliance rate of centralized drinking water supply in cities was 99.6% at the county level and above. The supply rate and population coverage rate of centralized drinking water supply in rural areas have reached more than 95%. Therefore, the main problem that the Zhejiang Province faces is air pollution. Referring to the existing pieces of literature [55,58], the regional air quality level was measured by the proportion of days when air quality reaches or is better than Grade II. It can reflect the changes in air pollution in the Zhejiang Province in a more accurate and long-term sequence.

#### 3.2.3. Data Source and Description

The data about the Environmental Performance Index (EPI) came from published websites (web addresses https://epi.yale.edu/epi-results/2020/country/chn, accessed on 2 February 2022). The other data came from “Zhejiang Statistical Yearbook” (2001–2020), Statistical Yearbook of various cities in Zhejiang (2001–2020), and “Zhejiang Statistical Data Compilation for the Past 60 Years”, and Statistical Bulletins of Zhejiang Province and regions (2000–2019). It should be pointed out that this paper calculated the per capita input and output according to the actual resident population, and selected the year-end resident population as the personnel base.

### 3.3. Methodology

#### 3.3.1. Efficiency Measurement

The current mainstream algorithms for efficiency are stochastic frontier analysis (SFA) and data envelopment analysis (DEA). SFA can only deal with the efficiency of a single output situation and needs to pre-set production functions and parameter values without considering the “unexpected output”, while traditional DEA models (such as BBC or CCR models) are based on radial angle, which can only be operated from an input or output perspective. Moreover, it does not take into account the slack problem of inputs and outputs, which often leads to the fact that the calculation results are not completely consistent with the actual situation. To make up for the shortcoming, the non-radial and non-angular SBM model was proposed by Tone (2001) [59], which directly incorporates the slack variables into the objective function. It can not only effectively solve the slack problem of inputs and outputs, but also ensure the accuracy of the calculation results after considering the unexpected outputs. It has been widely used in efficiency measurement and has shown good reliability [60]. Given the above, this paper also adopted the SBM model based on undesired outputs to measure the green economic efficiency and ecological welfare performance of cities in the Zhejiang Province.

Assuming that the city system has *n* decision-making units, each decision-making unit has three vectors, input *X*, expected output *Y^g^*, and undesired output *Y^b^*, and its elements can be expressed as $x_{i}$ ∈ *R^m^*, *y^g^* ∈ *R^S^*_1_, *y^b^* ∈ *R^S^*_2_. The matrices *X*, *Y^g^*, and *Y^b^* are defined as follows: *X* = [*x*_1_,…, *x_n_*] ∈ *R^m×n^*, $Y^{g}=\left[y_{1}^{g},\ldots {,y}_{n}^{g}\right]\;\in \;R^{s_{1}\times n},\mathrm{and}\;Y^{b}=\left[y_{1}^{b},\ldots ,y_{n}^{b}\right]\in R^{s_{2}\times n},$ where $x_{i}>0,y_{i}>0$ and $y_{i}^{b}>0\;\left(i=1,2,\ldots ,n\right)$. Then, the SBM efficiency measurement model can be expressed as:

(1) $$\rho =min\frac{1-\frac{1}{m}\sum_{i=1}^{m}\frac{s_{i}^{-}}{x_{i0}}}{1+\frac{1}{s_{1}+s_{2}}(\sum_{r=1}^{s_{1}}\frac{s_{r}^{g}}{y_{r0}^{g}}+\sum_{r=1}^{s_{2}}\frac{s_{r}^{b}}{y_{r0}^{b}})}$$

(2) $$s.t.\;\;\left\{\;\begin{array}{c} x0=X\lambda +s^{-}\;\;\;\;\;\;\;\;\;\;\;\;\;\;\;\;\;\;\;\;\;\;\;\;\;\;\;\;\;\;\;\;\;\;\;\;\\ \;y_{0}^{g}=Y^{g}\lambda -s^{g}\;\;\;\;\;\;\;\;\;\;\;\;\;\;\;\;\;\;\;\;\;\;\;\;\;\;\;\;\;\;\;\;\;\;\;\;\\ y_{0}^{b}=Y^{b}\lambda +s^{b}\;\;\;\;\;\;\;\;\;\;\;\;\;\;\;\;\;\;\;\;\;\;\;\;\;\;\;\;\;\;\;\;\;\;\;\;\\ s^{-}\geq 0,\;s^{g}\geq 0,\;s^{b}\geq 0,\;\lambda \geq 0\; \end{array}\right.\;\;\;\;\;\;\;\;\;\;\;\;\;\;\;$$

In the formulas, $s^{-}$, $S^{g},$ and $S^{b}$ represent the slack of input, expected output, and undesired output, respectively; and λ is the weight vector. The objective function ρ is strictly monotonically decreasing concerning $s^{-},s^{g}$, and $s^{b}$. When ρ = 1, that is, when $s^{-}$, $s^{g},$ and $s^{b}$ are all equal to 0, it indicates that the decision-making unit is efficient; if ρ < 1, it indicates that the decision-making unit has element redundancy, and the efficiency can be improved by optimizing the configuration.

#### 3.3.2. Modelling the Coupling Degree

Coupling refers to a phenomenon where two or more systems interact with each other. It originated from physics and gradually became an effective tool for analyzing the non-linear relationships among multiple factors [61]. Unlike concepts of correlation that refer chiefly to linear relationships [62], the concept of coupling generally describes the degree of interaction and influence amongst the elements of a system, or between different systems [63]. The coupling degree model is a typical application of hard science principles in the field of soft science. Because of its clear meaning and simple calculation, this model has been widely used in geographical research [64], urban studies, and environmental issues [63]. The expression of the coupling degree model generally has two forms. Given *n* ≥ 2 systems, *U_i_* ≥ 0 is used to represent the evaluation value of the system. The generalized calculation formula of the coupling degree can be expressed as Equations (3) and (4):

(3) $$C_{1}\left(U_{1},U_{2},\ldots ,U_{n}\right)=n\times {\left[\frac{U_{1}U_{2}\ldots U_{n}}{{\left(U_{1}+U_{2}+\ldots +U_{n}\right)}^{n}}\right]}^{\frac{1}{n}}$$

(4) $$C_{2}\left(U_{1},U_{2},\ldots ,U_{n}\right)=2\times {\left[\frac{U_{1}U_{2}\ldots U_{n}}{\prod_{i<j}{\left(U_{i}+U_{j}\right)}^{\frac{2}{n-1}}}\right]}^{\frac{1}{n}}$$

(5) $$D=\sqrt{CT},T=\alpha_{1}U_{1}+\alpha_{2}U_{2}+\ldots +\alpha_{n}U_{n}$$

In the equation, *C*_1_ and *C*_2_ indicate two kinds of coupling degrees. When *n* = 2, *C*_1_ = *C*_2_; when *n* > 2, 0 ≤ *C*_1_ ≤ *C*_2_ ≤ 1 [65]. D is the coupling coordination degree; *T* is the comprehensive coordination index; and *α*_1_, *α*_2_, …, *α_n_* are the undetermined coefficients. Regarding the existing research [66,67,68,69] and combined with the practical situation, this paper adopted *α*_1_ = *α*_2_ =, …, = *α_n_* = 1/*n*.

To date, there have been no uniform criteria for the coordination degree [70]. Many existing pieces of research have studied the green economic efficiency and ecological welfare performance in recent years, but most of them are limited within the scope of a single efficiency factor. There was little research on the coordination coupling degree between the two, whether it was based on the urban agglomeration or an individual city. To this end, referring to the previous studies [71], the coordination degree is classified into five categories as shown in Table 3.

#### 3.3.3. Spatial Econometric Approach

The spatial weight matrix is a prerequisite for spatial autocorrelation analysis, and it is also the basis for Moran’s I statistical tests and model construction. *W_ij_* represents the spatial weight, which is mainly divided into the spatial weight of adjacency relationship and the spatial weight of distance relationship. Considering the economic relations and practical requirements of the research, the minimum threshold distance of the spatial weight of distance relationship was chosen for this study.

(6) $$W_{ij}=\begin{array}{cc} \left\{\begin{array}{c} 1\\ 0 \end{array}\right. & \begin{array}{c} bound\;\left(i\right)\cap bound\;\left(j\right)\neq 0\\ bound\;\left(i\right)\cap bound\;\left(j\right)=0 \end{array} \end{array}$$

where *bound* (*i*) is the boundary of a spatial unit.

The Moran Index is divided into Global Moran’s I and Local Moran’s I. Global Moran’s I indicates whether there is agglomeration or anomaly in space, while local Moran’s I indicates where there is an anomaly or where there is agglomeration. The expression of Global Moran’s I is given by:

(7) $$I=\frac{N}{S_{0}}\frac{\sum_{i=1}^{N}\sum_{j=1}^{N}W_{ij}\left(y_{i}-\bar{Y}\right)\left(y_{j}-\bar{Y}\right)}{\sum_{i}^{N}{\left(y_{i}-\bar{Y}\right)}^{2}}$$

where $S_{0}=\sum_{i=1}^{N}\sum_{j=1}^{N}W_{ij}$, $\bar{Y}=\frac{1}{N}\sum_{y=1}^{N}y_{i}$, and $y_{i}$ represents the observed value of region *i*. *N* is the total number of observation regions. The expression of local Moran’s I is:

(8) $$I_{i}=\frac{Y_{i}-\bar{Y}}{S_{i}^{2}}\sum_{j=1,j\neq i}^{N}W_{ij}\left(Y_{j}-\bar{Y}\right)\;$$

Among them, the global spatial autocorrelation reflects the overall trend of spatial correlation in the entire region, and the local spatial autocorrelation reflects the spatial relationship among regions, both of which are measured by Moran’s I index.

## 4. Result Analysis

### 4.1. The Spatial and Temporal Evolution of Green Economic Efficiency and Ecological Welfare Performance of Cities in the Zhejiang Province

**Figure 3:**
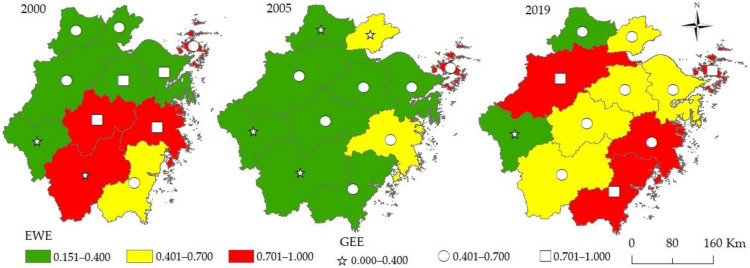
The spatial and temporal evolution of green economic efficiency and ecological welfare performance of cities in the Zhejiang Province.

Using a non-radial and non-angular SBM model with unexpected outputs, based on the panel data of 11 cities in the Zhejiang Province from 2000 to 2019, the green economic efficiency and the ecological welfare performance of each city in the Zhejiang Province were calculated. According to the calculation results, three time sections of 2000, 2005, and 2019 were selected. The efficiency value and spatial distribution of the two were further visualized with the help of ArcGIS 10.6 software (see Figure 3).

#### 4.1.1. Green Economic Efficiency

**Figure 4:**
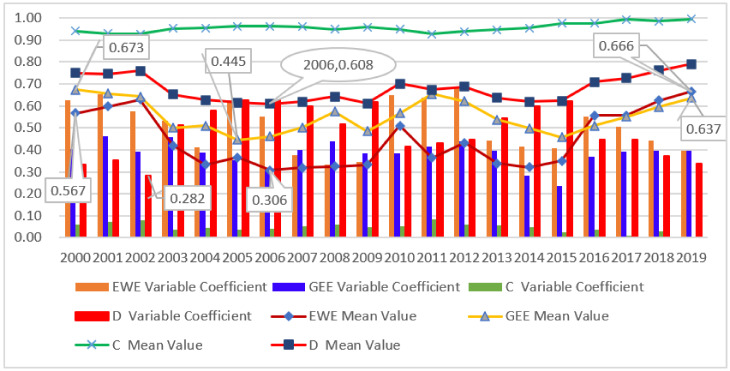
The dynamic evolution trend of green economic efficiency, ecological welfare performance, coupling degree, and coupling coordination degree.

From Figure 3 and Figure 4, it can be seen that the overall green economic efficiency shows a wave-like advancement and the gaps between the 11 cities were eased. Specifically, the green economy efficiency of each city fluctuated greatly in the research period. In 2005, no city was in the advanced coordination stage (D5). It also can be seen from Figure 4 that the average green economic efficiency of the Zhejiang Province has remained relatively stable as a whole, ranging from 0.673 in 2000 to 0.637 in 2019, reaching the lowest point of 0.445 in 2005. Judging from the development gap between each city over the years, the coefficient of variation reached a maximum value of 0.459 in 2003 and a trough of 0.234 in 2015, showing a wave-like downward trend as a whole. Five cities entered a higher stage to varying degrees, two cities declined (namely Jinhua and Lishui), and four cities remained unchanged, staying at the same stage at both the beginning and end of the study.

#### 4.1.2. Ecological Welfare Performance

Figure 4 shows that the ecological welfare performance presents a downward trend [38] and the periodic characteristics of “first decline and then rise” [50], which is consistent with the existing research. However, after reaching its lowest point in 2006, it showed an overall upward trend in the “U” shape. Its average value decreased from 0.567 in 2000 to the lowest point of 0.306 in 2006 and then increased rapidly, reaching 0.510 in 2010 and 0.666 in 2019, an increase of 17.56% and an amplitude of 117.62% in the study period. It can also be seen from Figure 3 that five cities entered a higher stage to varying degrees, two cities declined (namely Jinhua and Lishui), and four cities remained unchanged, staying at the same stage at both the beginning and end of the study. Zhoushan was in a state of good welfare performance during the whole study period. In 2005, Taizhou and Jiaxing were in the second stage, and the rest were in the first stage.

### 4.2. Dynamic Evolution Trend of the Coupling Coordination Degree between Green Economic Efficiency and Ecological Welfare Performance of Cities in Zhejiang Province

#### 4.2.1. Temporal Variation of Coupling Degree and Coupling Coordination Degree

From a macro view, the overall coupling degree between green economic efficiency and ecological welfare performance maintains a relatively high level, and it still shows a wave-like upward trend in an overall stable state, and it is generally higher than the initial value. It can be seen from Figure 4 that the mean coupling degree fluctuates in [0.92, 0.99]. At the same time, the amplitude was within 7.7% in the study period, which indicated the stability of the coupling degree development from a side. It can also be found from Figure 4 that the mean value of the coupling coordination degree between the two systems presents a “U”-shaped development trend. It reached the lowest point of 0.608 in 2006, and the amplitude reached 30.2%, which is nearly four times that of the coupling degree. We can see that there is a relatively stable and strong interaction between the two systems. Therefore, there is a relatively stable interaction mechanism between green economic efficiency and ecological welfare performance, and the coordination degree can better show the development trend of their coordinated development.

#### 4.2.2. Differences in Coupling Coordination

To explore the difference in coupling coordination degree among 11 cities in the Zhejiang Province, the coefficient of variation was used to reveal it. The coefficient of variation can objectively reflect the degree of difference within a set of data. Compared with indicators such as range, variance, and standard deviation, it has the advantage of more accurately reflecting the degree of data dispersion [72]. With the help of the coefficient of variation formula, the year-by-year differences between cities are calculated and drawn into the distribution diagram shown in Figure 4. Among them, the smaller the coefficient of variation, the smaller the difference in the indicator value among cities. It can be seen from Figure 4 that the coefficient of variation presents a wave-like development trend and has declined year by year in recent years. Specifically, in the entire study period ranging from 0.334 in 2000 to 0.335 in 2019, the difference reached a minimum of 0.282 in 2002 and a maximum of 0.628 in 2005. Throughout the study period, the peaks occurred in 2005, 2009, and 2015. In 2009, it rebounded rapidly and reached a stage high point. After a rapid return to the stage bottom point in 2010, it rebounded slowly and reached the second peak of 0.622 in 2015, and then began to decline obviously. The obvious downward trend means that the differences among cities were decreasing significantly.

#### 4.2.3. Spatial and Temporal Development Characteristics of Coupling Coordination Degree

Based on the framework of distributed dynamics and referring to the practice of existing studies [28,50], the coupling coordination degree is mainly divided into five types, General Disorder (D1), Preliminary Disorder (D2), Preliminary Coordination (D3), Moderate Coordination (D4), and Advanced Coordination (D5) in Table 3.

**Figure 5:**
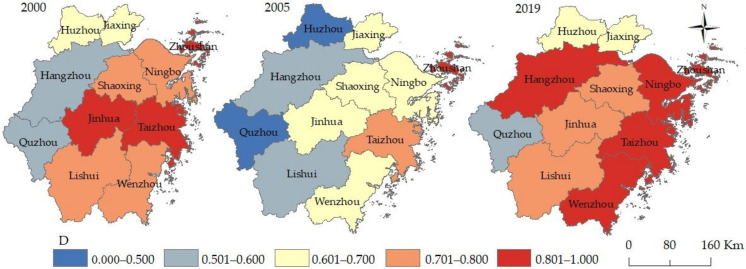
The spatiotemporal development characteristics of the coupling coordination degree.

Figure 5 presents a vector diagram of the spatiotemporal development characteristics of the coupling coordination degree in 2000, 2006, and 2019. From the overall picture, the number of Advanced Coordination (D5) cities changed from two in 2000 to two in 2019; the number of Moderate Coordination (D4) cities changed from the original four to three; and the Preliminary Coordination (D3) cities remained unchanged, still the original Huzhou and Jiaxing. From the perspective of specific cities, Zhoushan and Jiaxing remained unchanged throughout the study period, and they were, respectively, in Advanced Coordination (D5) and Preliminary Coordination (D3). Huzhou, Quzhou, Lishui, Shaoxing, and Taizhou all stayed at the same stage at both the beginning and end of the study. All these cities experienced a setback of at least one stage during the study period. Among them, Huzhou fell from Preliminary Coordination (D3) to General Disorder (D1), and Lishui descended from Moderate Coordination (D4) to Preliminary Disorder (D2). During the study period, Hangzhou improved significantly, jumping from Preliminary Disorder (D2) to Advanced Coordination (D5), and Ningbo and Wenzhou entered the rank of Advanced Coordination (D5) from Moderate Coordination (D4). Jinhua was the only city that retreated in the study period, from Advanced Coordination (D5) in 2000, to Preliminary Coordination (D3) in 2005, and then slowly rose, reaching Moderate Coordination (D4) in 2019. The reasons for Jinhua’s backwardness are multi-faceted, and it is the result of the combined effect of multiple factors, such as the economy, society, and the natural environment.

#### 4.2.4. Spatial and Temporal Correlation Characteristics of the Coupling Coordination Degree

**Figure 6:**
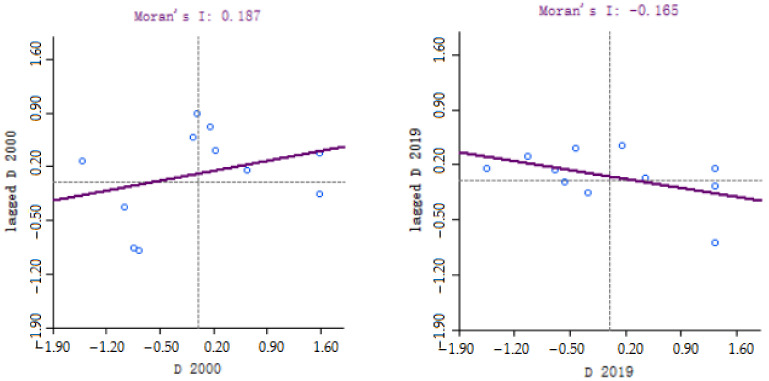
Moran’s I scatter plot for the coupling coordination degree of each city in 2000 and 2019 (the line represents the global Moran’s I index for the year. Each dot represents the location of a sample point. The distance of the dot from the origin in the figure represents the level of aggregation significance, and the farther away from the origin, the better the significance level).

**Figure 7:**
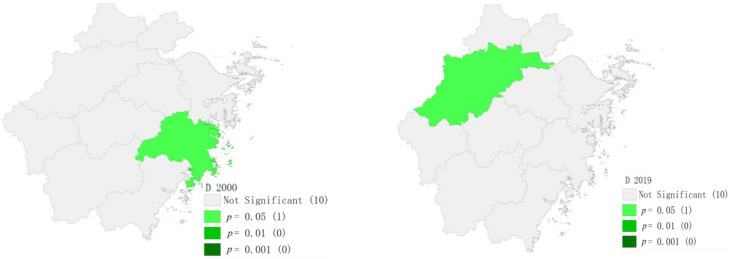
Local spatial correlation map of cities in the Zhejiang Province in 2000 and 2019.

Moran’s I is a statistic for testing spatial autocorrelation, which can reflect the correlation degree and spatial distribution pattern of cities in Zhejiang and neighboring regions. Its value is in the interval [−1, 1]. If it is less than 0, it means a negative correlation, and there is a spatial dispersion feature; if it is greater than 0, it means a positive correlation, and there is a spatial aggregation feature [28]. Based on the coupling coordination degree of cities in the Zhejiang Province in 2000 and 2019, Moran’s I was calculated by an adjacency matrix, as shown in Figure 6. The results show that the Moran index is positive and passes the significance test, indicating that the coupling coordination degree between the ecological welfare performance and green economic efficiency of the cities in Zhejiang has a positive spatial correlation, and the spatial aggregation ability is strong. This means that the higher the coupling coordination degree of a certain region, the more likely it is to connect with regions with a high coupling degree. Conversely, the lower the region, the easier it is to gather around the regions with a low coupling degree. The local Moran index can explore the spatial aggregation pattern of the coupling coordination degree between the ecological welfare performance and green economic efficiency in the Zhejiang Province. From the scatter plot in Figure 6 and the local spatial correlation diagram in Figure 7, it can be seen that the Moran index in 2000 was positive, with High–High (first quadrant) and Low–Low (third quadrant) being dominant. Taizhou, which is in High–High, passed the significance test. In 2019, Moran’s I became negative, mainly concentrated in Low–High (second quadrant) and High–Low (fourth quadrant), and Hangzhou, which is in High–Low, passed the significance test.

From Table 4, it can be found that there is a large difference in the Moran index over the years, and the spatial autocorrelation passed the test of 95% and above except in 2016. Therefore, it has spatial autocorrelation, which is a mainly positive correlation. In the past three years, the coordination degree of Hangzhou was relatively high and significantly higher than that of neighboring regions. Especially, the neighboring Quzhou was still at the Preliminary Disorder (D2) level, which formed a clear phenomenon of High–Low and Low–High agglomeration. It can be found that the regional spatial spillover effect of the coordinated development between green economic efficiency and ecological welfare performance was significantly weaker than its internal development factors.

## 5. Analysis of the Influencing Factors of the Coupling Coordination Degree between Green Economic Efficiency and Ecological Welfare Performance

### 5.1. Variables

To further explore the influencing factors of the coupling coordination degree between green economic efficiency and ecological welfare performance, this paper drew on the research results of related scholars [17,50,73]. Referring to the research on green economic development, ecological welfare performance, and ecological civilization construction, this paper follows the characteristics of China’s economic and social development and the principles of data accuracy and availability. Starting from the factors of regional urbanization, industrialization development level, industrial structure, government support, opening to the outside world and economic development, innovation ability, and Internet development, this paper explored their influences on the coupling coordination degree between green economic efficiency and ecological welfare performance. The indicator variables are listed in Table 5.

### 5.2. Model Introduction and Result Analysis

Since the coupling coordination degree between green economic efficiency and ecological welfare performance is in the interval [0, 1], which is a “restricted dependent variable”, the Tobit model can solve the problem of restricted dependent variables well [74,75,76]. Therefore, this paper selected the Tobit model to explore the factors that may affect the coordinated development of ecological welfare performance and green economic efficiency. Based on the panel data of 11 prefecture-level cities in the Zhejiang Province from 2000 to 2019, the econometric model was constructed as follows:

(9) $$D_{it}=cons+X_{it}{}^{\prime }\beta +\varepsilon_{it}\;,\;i=1,2,\cdots ,11\;;\;t=1,2,\cdots ,20\;$$

$$X_{it}=X_{1it},X_{2it},\cdots ,X_{kit}\;\beta =\beta_{1},\beta_{2},\cdots ,\beta_{k}$$

In this formula, *cons* is a constant term, $X_{it}$ and β are k-dimensional column vectors, and $\varepsilon_{\mathrm{it}}$ is a random disturbance term. This paper used Stata17.0 to obtain the parameter results, the significance level of the regression equation was 0.000, and the equation as a whole passed the significance test. It can be seen from Table 6 that the proportion of the urban population and the coupling coordination degree have a significant positive relationship, and the coefficient of the relevant variable is relatively large, indicating that urbanization development can promote the coupling coordination degree. The proportion of total industrial output value in GDP has a significant negative impact on the coupling coordination degree. Although the high energy consumption and high pollution of the industry can increase GDP, it may not simultaneously promote the green efficiency of the environment, especially the improvement of ecological welfare. The proportion of the output value of the tertiary industry in GDP has a significant positive effect on the coupling coordination degree, and the further rational optimization and transformation and upgrading of the industrial structure can also contribute to the improvement of the coupling coordination degree between green economic efficiency and ecological welfare performance. The regression coefficient of the proportion of local fiscal expenditure in GDP is 0.00871 and is significant. It has a large positive impact on the coupling coordination degree, indicating that the government’s macro-control can turn ecological advantages into a latecomer advantage of economic development, and is conducive to improving regional ecological welfare and green economy. The actual utilization of foreign capital per capita is significantly negative, indicating that foreign investment cannot promote the coordinated development of ecological welfare performance and green economic efficiency. The regression coefficient of GDP per capita has turned from positive in Equation (6) to negative in Equation (7), and the regression coefficient of the squared GDP per capita is positive, but both of them are not significant, indicating that the economic growth cannot necessarily promote the coordinated development of the two. Similarly, the influence of regional innovation ability and network popularization on the coupling coordination degree is not obvious and failed to pass the significance test.

### 5.3. Robustness Test

Referring to the previous research and the usual test methods [76,77,78,79], this paper used two methods of omitted variable test and substitution test to test the robustness of the Tobit regression results.

#### 5.3.1. Omitted Variable Test

The level of environmental protection may also have an impact on the regression results and is a possible omitted variable in this paper. Accordingly, the comprehensive utilization rate of industrial solid waste is used to measure the level of environmental protection and is added to the model for re-regression. The results are listed in Table 7 (1) and (2). After adding the comprehensive utilization rate of industrial solid waste (utir), the results are still consistent with the Tobit regression results in Table 6.

#### 5.3.2. Substitution Test

The industrial structure in Table 6 is measured by the proportion of the output value of the tertiary industry in GDP, but some scholars believe that the proportion of the output value of the secondary and tertiary industries in GDP can also represent the industrial structure development. Therefore, next, we used the proportion of the output value of the secondary and tertiary industries in GDP as an alternative indicator (ind23), and the regression was performed again. The results are also consistent with the Tobit regression results in Table 6. The specific regression results are listed in (3) and (4) of Table 7. Therefore, the equation passes the omitted variable test and the substitution test, and the Tobit model is suitable and reliable.

## 6. Discussion

### 6.1. Green Economic Efficiency (GEE) Can Promote Ecological Welfare Performance (EWP) and There Is a Synergistic Effect between the Two System

Traditional economic growth only emphasizes economic output while ignoring its negative effects on the environment and resources [14]. The green economy is guided by the concept of green development, introduces environmental and resource factors [80], and pays attention to the good coordination of the environment and economy [81]. The efficiency-oriented green economy theory improves economic efficiency through green development. While promoting stable economic growth, it reduces resource consumption and pollution emissions to achieve sustainable economic development. It is more reasonable than the way that only considers the input of production factors in the past [47]. The research on ecological welfare performance can be traced back to Daly [82] who proposed that the sustainable development level of each country can be evaluated by measuring the social welfare level generated per unit of natural resource consumption, but Daly [82] did not give any practical specific quantitative indicators. The Chinese scholar Zhu [83] first proposed the concept of ecological welfare performance in 2008 based on Daly’s [82] thoughts, defined it as the efficiency of converting ecological resource consumption into social welfare level, and quantified it by the ratio of human development index to ecological footprint, which started the studies in China on ecological welfare performance. Ecological welfare performance refers to the efficiency of converting ecological input into social welfare level, which is proposed under the framework of sustainable development economics. As in the case of neoclassical economics, it believes that natural capital and artificial capital can be replaced by each other. The development of the green economy is conducive to promoting economic development and green benefits, which will promote regional economic development and provide strong economic support for regional education and medical care. The improvement of green benefits reduces the discharge of regional pollutants, thereby further promoting the environmental quality level. Therefore, the improvement of green efficiency has a promoting effect on ecological welfare performance, and the two have a mutually promoting and coordinated relationship. The improvement of ecological welfare can also promote energy utilization efficiency and the environment, thereby forcing the transformation and upgrading of regional economic development and the further rational layout of the industrial structure. At the same time, the improvement of welfare also requires sound economic development and social demand for high-quality medical care. The improvement of ecological welfare can promote human capital, which in turn promotes green economic efficiency. As a result, there is a coupling relationship between the two. It can be verified from Figure 4 that the coupling effect between the two always maintains a high level of about 0.9. It is well proven both theoretically and empirically.

### 6.2. Does Economic Growth Necessarily Promote Green Economy and Ecological Welfare Performance?

China’s economy has been growing rapidly for nearly 40 years, with the GDP per capita reaching USD 11,300 in 2020 from USD 0.2 million in 1978, and its economic aggregate has surpassed Japan since 2010 to become the second largest in the world. However, its Environmental Performance Index only ranks 120th out of 180 countries (2020). It has been proved that economic development does not mean that the environment can also improve simultaneously. Some scholars have pointed out that there is an inverted “U”-shaped relationship between economic growth and ecological welfare performance [41]. The relationship between economic growth and green economy is mainly reflected in the relationship between economic growth and pollution and the relationship between economic agglomeration and green economic development. The former mainly has the famous Kuznets curve, while for the latter some scholars have also drawn some conclusions: when the degree of economic agglomeration is reasonable, its impact on green economic efficiency is positive (mainly showing agglomeration effect); when the degree of economic agglomeration is above the critical value, the impact is negative (mainly showing congestion effect) [84]. Therefore, economic development has uncertainties on ecological welfare and green economic efficiency. It can also be verified from the research on the influencing factors in this paper that the impact of economic growth on the coordinated development of green economic efficiency and ecological welfare performance is not obvious. To verify that there is a nonlinear relationship, the square of GDP per capita is introduced, and the result is still insignificant. Therefore, the existing facts and model evidence confirmed each other that economic development may not necessarily promote the coordinated development of green economic efficiency and ecological welfare performance. Both linear and quadratic relationships failed the significance test.

### 6.3. Does the “Two Mountains” Theory Really Work?

On 15 August 2005, Xi Jinping went to Yucun village for an investigation. In a small conference room in the village, Bao Xinmin, the then secretary of the village party branch, reported that Yucun Village had adopted a democratic decision to shut down the mines that polluted the environment, and started to engage in eco-tourism intending to allow villagers to make money from the scenery. President Xi Jinping said: “You must stop thinking about going the old way and still being so obsessed with the old development model. So, as you said just now, it is a wise move to make up your mind to close some mines. lucid waters and lush mountains are invaluable assets. We used to say that we need only not lucid waters and lush mountains, but also invaluable assets. Lucid waters and lush mountains are invaluable assets”. This is the first time President Xi Jinping has put forward the ecological and environmental protection concept of “lucid waters and lush mountains are invaluable assets”, namely, the “Two Mountains” theory. Then, it explained the dialectical and unified relationship between economic development and environmental protection from different perspectives [3] and answered a series of major theoretical and practical questions, such as what is ecological civilization and how to build an ecological civilization, providing scientific guidance for building a beautiful China. From the development background and substantive connotation of the “Two Mountains” theory, it can be seen that, first, the concept of ecological environmental protection is emphasized, which is consistent with the essence of green economic development. Second, what is emphasized is shutting down high-input, high-output, and high-polluting enterprises, and developing tertiary industries, such as eco-tourism. This is essentially a transformation and upgrading of the economic structure. Theoretically, the importance of economic structural transformation and upgrading is emphasized. In terms of actual development, the economic structure plays a greater role in promoting regional green economic efficiency and ecological welfare performance, while industrialization plays a negative role. From the perspective of historical development, after the “Two Mountains” theory was put forward in Zhejiang, Zhejiang’s overall green economic efficiency and ecological welfare performance reached inflexion points in 2005 and 2006, respectively, and were at the lowest level in the entire study period (see Figure 4). Finally, due to the importance attached by the Chinese party and government to the “Two Mountains” theory, they will inevitably be inclined towards and give support to relevant policies and other aspects. As long as the government attaches great importance to it, it can concentrate its superior forces to do great things. China’s good control of the COVID-19 epidemic is an important manifestation of its system advantages in this regard. It can concentrate national strength to do important things, and other things must serve the organization’s major affairs. The facts also proved that the investment of the national government can be conducive to the coordinated development of green economic efficiency and ecological welfare performance. In a broad sense, the “Two Mountains” theory is a comprehensive development theory with Chinese characteristics that integrates the transformation and upgrading of economic structure and the strong support of the state, regarding the implementation of ecological and environmental protection as important as the economy. Therefore, the “Two Mountains” theory does promote the coordinated development of green economic efficiency and ecological welfare performance.

## 7. Conclusions and Policy Implication

### 7.1. Conclusions

Through the above analysis and discussion, this paper draws the following important conclusions:

(1) The average green economic efficiency in the Zhejiang Province experienced a “U”-shaped wave-like development process that first decreased and then increased, reached the lowest point of the entire research period in 2005, and remained at the same level at both the beginning and end of the research period. From the perspective of the coefficient of variation, the gaps between various regions also change in the manner of a wave with an overall trend of becoming more stable and smaller.

(2) The overall mean value of ecological welfare performance in the Zhejiang Province moves in a “U” shape similar to that of green economic efficiency, but it is flatter. The inflexion point appeared in 2006, and it was significantly higher at the end of the study than at the beginning of the study, by an overall increase of 17.5%. From the perspective of the coefficient of variation, the gaps between regions are still very obvious, and the mutation phenomenon is relatively obvious. Fortunately, in the last 4 years of the study period, there was moderation and decrease.

(3) The overall mean of the coupling effect between green economic efficiency and ecological welfare performance of 11 cities in the Zhejiang Province remained unchanged. The coupling coordination degree decreased first, reaching the lowest point in 2006, and then rose in large waves mixed with small waves. Regarding the distribution characteristics of coefficient of variation, it generally presents an inverted “U” shape and has a stabilizing trend. In terms of the spatial development characteristics of each prefecture-level city, except for Jinhua, all other cities are developing towards more advanced levels, and some places, such as Hangzhou, turned from Preliminary Disorder (D2) at the beginning into the highest level of Advanced Coordination (D5) at the end of the study period. According to the spatial correlation, a turning point can be found in 2016. Previously, it mainly showed a positive correlation of High–High and Low–Low agglomeration, and later became a spatially dispersed High–Low agglomeration, which proved that self-development is the main reason for coordinated development.

(4) Among the driving factors of the coupling coordination degree, the urbanization level, industrial structure, and government investment play a role in promoting the coordinated development of the two regional systems, while the industrialization degree and the level of opening to the outside world hurt it. The impact of economic development, innovation capacity, and Internet development on regional coordinated development is not significant.

(5) The “Two Mountains” theory is beneficial to the improvement of regional green economic efficiency and ecological welfare performance from both theoretical analysis and practice and has a positive effect on the coordinated development of the two, which has been verified empirically.

### 7.2. Policy Implication

Based on the above conclusions and the actual situation of various cities in the Zhejiang Province, this paper puts forward policy recommendations to promote the in-depth coupling coordination between green economic efficiency and ecological welfare performance in various regions:

(1) The effects of green development benefits and ecological civilization emphasized by the “Two Mountains” theory were shown. In 2005, the “Two Mountains” theory was first clearly put forward and put into practice in Zhejiang. The green economy efficiency reached the lowest point in 2005 and then rose in waves. The ecological welfare performance reached the lowest point in 2006. After that, it also gradually recovered with a callback, showing an overall upward trend. This shows that, under the influence of the policy, the economy can be developed, while “greening” and ecological welfare can be improved. Therefore, all localities must deeply understand the “Two Mountains” theory and implement it in economic development in a good way.

(2) Take the high-quality development strategy and the Common Prosperity Demonstration Zone as an opportunity to accelerate the optimization of industrial layout and the transformation and upgrading of industrial structure, reduce pollutant emissions, and improve economic efficiency, aiming to achieve a new model of green and sustainable development [85]. It is proposed to improve the shortcomings of various regions, further develop the green economy while improving the efficiency, further optimize the ecology while improving the welfare, thereby enhancing the ecological welfare performance and green economic efficiency, and ultimately achieve a better and more satisfying life for the people. It also sets up a template for the “Common Prosperity Demonstration Zone” and provides a successful experience for the whole country.

(3) Guided by comprehensively promoting the coupling coordination, it is proposed to make full use of big data and other informatization means, seize the good opportunities of digital economy development, innovate and cultivate new economic growth poles, especially in the ecological field, and unleash the high kinetic energy of society and nature, to boost the realization of high-level coordinated development of the two systems.

From the perspective of prefecture-level cities, this paper is the first to use the undesirable SBM model to measure the green economic efficiency and ecological welfare performance of 11 prefecture-level cities in the Zhejiang Province, China. The calculated efficiency value is a relative one for a region, not an absolute one, and the results are affected by the selection of regions and input-output variables to a certain extent. In the future, it can be further refined to the county-level research, as well as the comparative study of the “Two Mountains” theory to further explore the mechanism of the “Two Mountains” theory.

## Figures and Tables

**Table 1:** Green economy efficiency input–output index system.

Category	First-Level Indicators	Second-Level Indicators	Attribute Description	Unit
Input indicators	Energy input	Industrial electricity consumption		10^4^ kw·h
	Labor input	Employees of the whole society	The total number of employees in the primary, secondary, and tertiary industries.	10^4^ persons
	Capital investment	Capital stock	The capital stock of each region is calculated by perpetual inventory method. Please refer to the processing methods of Boya Li [19].	CNY 10^8^
Output indicators	Expected output	Regional GDP	(+)	CNY 10^8^
	Undesired output	Industrial wastewater discharge	(−)	10^4^ t
		Industrial SO_2_ emissions	(−)	t
		Industrial smoke (powder) and dust emissions	(−)	t

Note: “+” represents the desired output, the bigger the better; “−” represents the undesired output, the smaller the better.

**Table 2:** Input–output index system of ecological welfare performance.

Category	First-Level Indicators	Second-Level Indicators	Third-Level Indicators/Description	Unit
Ecological input indicators	Resource consumption	Energy consumption	Industrial electricity input per capita	kw·h/person
		Land consumption	Built-up land area per capita	M^2^/person
		Water consumption	Water resources per capita	M^3^/person
	Environmental pollution	Wastewater disposal	Wastewater disposal per capita	t/person
		Exhaust emissions	SO_2_ emissions per capita	t/person
		Solid waste discharge	Industrial solid smoke (powder) emissions per capita	t/person
Welfare output indicators	Social welfare level	Economic development level	GDP per capita (+)	10^4^ CNY/person
		Educational development level	Average years of education (+)	Year
		Health care level	Number of beds per 10,000 people (+)	beds/10^4^ person
		Air quality level	The proportion of days when air quality reaches or is better than grade II (+)	(%)

Note: Average years of education = (6 × P primary school + 9 × P junior high school + 12 × P high school + 16 × P college or above)/(P primary school + P junior high school + P high school + P college or above), where P means a person; “+” represents the desired output, the bigger the better.

**Table 3:** Classification criteria for coordination.

Coordination Interval	Coordination Level	Symbol
0.0 ≤ D < 0.5	General disorder	D1
0.5 ≤ D < 0.6	Preliminary disorder	D2
0.6 ≤ D < 0.7	Preliminary coordination	D3
0.7 ≤ D < 0.8	Moderate coordination	D4
0.8 ≤ D ≤ 1.0	Advanced coordination	D5

**Table 4:** Moran index values and regions passing the significance test over the years.

Year	Moran’s I	*p*	High–High	High–Low	Low–Low	Low–High
2000	0.187	0.05	Taizhou			
2001	−0.078	0.05		Jiaxing		
2002	0.243	0.05		Jiaxing	Hangzhou *	
2003	0.192	0.05	Ningbo			
2004	0.236	0.01	Ningbo			
2005	0.179	0.01	Ningbo			
2006	0.068	0.01				Ningbo
2007	0.254	0.01	Ningbo			
2008	0.221	0.05	Ningbo			
2009	0.188	0.05		Hangzhou		
2010	0.065	0.05			Quzhou	
2011	0.022	0.05	Taizhou			
2012	0.168	0.01		Ningbo		
2013	0.343	0.05	Ningbo			
2014	0.283	0.05	Ningbo			
2015	0.187	0.05	Ningbo	Hangzhou		
2016	−0.051	0.05				
2017	−0.267	0.05		Hangzhou		
2018	−0.083	0.05		Hangzhou		
2019	−0.165	0.05		Hangzhou		

* *p* < 0.01.

**Table 5:** Variables for the influencing factors of coupling coordination degree.

Variable	First-Level Indicators	Second-Level Indicators	Description and References	Symbol
Dependent variable	Coordinated development	Coupling coordination degree	Describe the coordinated development level of GEE and EWP	*D*
Independent variables	Urbanization	The proportion of the urban population	Urbanization level	*urb*
	Industrialization	The proportion of total industrial output value in GDP	Industrialization level	*ind*
	Industrial structure	The proportion of the output value of the tertiary industry in GDP	The rationality of industrial structure and structural transformation and upgrading	*ter*
	Government input	The proportion of local fiscal expenditure in GDP	Government support level	*gov*
	Opening to the outside world	The actual utilization of foreign capital per capita	The logarithm of the actual utilization of foreign capital per capita	*open1*
	Economic development	GDP per capita	Regional economic development level	*pgdp*
		The square of GDP per capita	Examine its nonlinear relationship with the coordination degree	*pgdp2*
	Innovation capacity	Patent applications per 10,000 people	Reflect regional innovation capabilities	*inv*
	Internet development	Accounts per 10,000 people	Reflect the popularity of networking	*int*

**Table 6:** Tobit regression results.

Var	(1)	(2)	(3)	(4)	(5)	(6)	(7)
*urb*	0.00502 ***	0.00645 ***	0.0073415 ***	0.00657 ***	0.00728 ***	0.00668 ***	0.00870 ***
	[0.000792]	[0.000699]	[0.0008217]	−0.000747	[0.000830]	[0.001074]	[0.001171]
*ind*		−0.00139 ***	−0.0012709 ***	−0.00130 ***	−0.00115 ***	−0.00116 ***	−0.00115 ***
		[0.000158]	[0.0001671]	[0.000197]	[0.000211]	[0.000212]	[0.000220]
*ter*			0.0032431 **	0.00303 **	0.00322 **	0.00306 **	0.00455 **
			[0.0016138]	[0.001508]	[0.001499]	[0.001507]	[0.001571]
*gov*				0.00788 ***	0.00817 ***	0.00871 ***	0.0071494 ***
				[0.001692]	[0.001686]	[0.001792]	[0.0016285]
*open1*					−0.0000914 *	−0.000123 **	−0.0001633 ***
					[0.000048]	[0.00006]	[0.00006]
*pgdp*						0.00384	−0.0021
						[0.004383]	[0.011426]
*pgdp2*							0.00051
							[0.000722]
cons	0.391 ***	0.509 ***	0.742 ***	0.730 ***	0.696 ***	0.716 ***	0.870 ***
	[0.046127]	[0.041821]	[0.1229662]	[0.095213]	[0.096083]	[0.098685]	[0.131002]
var(e.d)	0.0155 ***	0.0114 ***	0.0112 ***	0.0104 ***	0.0102 ***	0.0102 ***	0.0099 ***
	[0.001488]	[0.001098]	[0.001077]	[0.001]	[0.000984]	[0.00098]	[0.000949]
Log likely-hood	141.80249	175.02862	177.02849	184.7094	186.50245	186.8854	190.94897

Standard errors in parentheses; * *p* < 0.05, ** *p* < 0.01, *** *p* < 0.001.

**Table 7:** Tobit robustness test results.

	(1)	(2)	(3)	(4)
*urb*	0.00687 ***	0.00748 ***	0.00847 ***	0.00943 ***
	(0.001131)	(0.000819)	(0.001156)	(0.000906)
*ind*	−0.00102 ***	−0.00107 ***	−0.00114 ***	−0.00118 ***
	(0.000243)	(0.000210)	(0.000217)	(0.000178)
*ter*	0.00285 *	0.00315 *		
	(0.001486)	(0.001475)		
*gov*	0.00799 ***	0.00758 ***	0.00706 ***	0.00608 ***
	(0.001860)	(0.001673)	(0.001604)	(0.001339)
*open1*	−0.000148 *	−0.0000926 *	−0.000165 **	−0.000102 *
	(0.000063)	(0.000047)	(0.000062)	(0.000047)
*pgdp*	−0.000272		0.00400	
	(0.011513)		(0.011493)	
*gdp2*	0.000380		0.000182	
	(0.000724)		(0.000722)	
*utir*	−0.00172 **	−0.00169 **	−0.00165 *	−0.00157 *
	(0.000648)	(0.000635)	(0.000639)	(0.000629)
*ind23*			0.00443 **	0.00438 **
			(0.001547)	(0.001529)
cons	0.835 ***	0.818 ***	1.002 ***	0.945 ***
	(0.113842)	(0.104959)	(0.138773)	(0.124078)
var(e.d)	0.00980 ***	0.00988 ***	0.00958 ***	0.00969 ***
	(0.000944)	(0.000952)	(0.000920)	(0.000931)
Log likelihood	190.88721	190.00062	194.23315	192.95838

Standard errors in parentheses; * *p* < 0.05, ** *p* < 0.01, *** *p* < 0.001.

## Data Availability

Not applicable.
